# CHANGES IN FUNCTIONAL DISABILITY AND SUICIDAL IDEATION ASSOCIATED WITH PSYCHOTHERAPY FOR DEPRESSION

**DOI:** 10.1093/geroni/igz038.3041

**Published:** 2019-11-08

**Authors:** Julie Lutz, Sherry Beaudreau, Ruth Morin, David Bickford, J Craig Nelson, Scott Mackin

**Affiliations:** 1 West Virginia University, Morgantown, West Virginia, United States; 2 Sierra Pacific Mental Illness Research Education and Clinical Center, VA Palo Alto Health Care System, Palo Alto, California, United States; 3 San Francisco VA Medical Center, San Francisco, California, United States; 4 University of California, San Francisco Department of Psychiatry, San Francisco, California, United States

## Abstract

Given the association between functional disability and suicidal ideation (SI) in late life (Lutz & Fiske, 2017), this study examined associations between functional disability and SI among older adults receiving problem solving therapy (PST) for depression. PST is a promising intervention for SI (e.g., Gustavson et al., 2016) and has been shown to be effective in reducing functional disability among older adults with depression (e.g., Choi et al., 2014). Regression analyses with adults age 65-91 (n=65) found that level of SI (Geriatric Suicide Ideation Scale) at baseline was not significantly associated with change in SI from pre- to post-treatment. However, lower baseline disability (WHODAS-II; ΔR2=.08, p=.022) and greater reduction in disability (ΔR2=.12, p=.004) were significantly associated with greater reduction in SI after controlling for age and baseline SI. These results suggest that treatments that decrease disability may be particularly advantageous for reducing SI in older depressed adults.

